# Supplementary material for: Food Neophobia in Children with Autistic Spectrum Disorder (ASD): A Nationwide Study in Brazil
Source: Children (Basel). 2022 Dec 6;9(12):1907. doi: 10.3390/children9121907 (PMC9776952; doi:10.3390/children9121907)
Supplement: Supplementary file 1 [file children-09-01907-s001.zip › children-2082554-supplementary.pdf]

Table S1. Characterization of ASD caregivers (n=593). Brazil, 2020-2021.

	**Categories**	**Sample**	
		**n**	**%**
**Caregivers gender**	Male	28	4.7%
	Female	565	95.3%
**Degree of kinship**	Mother	544	91.7%
	Father	24	4.0%
	Grandparents	11	1.9%
	Brothers and sisters	4	0.7%
	Uncles	6	1.0%
	Cousin	2	0.3%
	No relationship	2	0.3%
**Marital status**	Single	101	17.0%
	Married/ stable union	411	69.3%
	Separate / divorced	71	12.0%
	Widower	10	1.7%
**Schooling**	from 1st to 4th grade of elementary school (former primary)	8	1.3%
	From 5th to 8th grade of elementary school (former gym)	20	3.4%
	High School (2nd degree) incomplete	30	5.1%
	Complete high school	163	27.5%
	Higher Education Incomplete	93	15.7%
	Higher Education Complete	113	19.1%
	Postgraduate	128	21.6%
	Master's degree	31	5.2%
	Doctorate degree	4	0.7%
	Postdoctoral	3	0.5%
**Monthly family income**	No income	32	5.4%
	Up to 1 MW	128	21.6%
	up to 2 MW	111	18.7%
	up to 3 MW	70	11.8%
	up to 4 MW	35	5.9%
	up to 5 MW	36	6.1%
	up to 6 MW	19	3.2%
	up to 7 MW	18	3.0%
	up to 8 MW	15	2.5%
	up to 9 MW	17	2.9%
	Between 10 and 12 MW	34	5.7%
	Between 13 and 15 MW	12	2.0%
	Above 15 MW	31	5.2%
	Not informed	35	5.9%

Table S2. Characterization of ASD children (n=593). Brazil, 2020-2021.

	Categories	Sample	
		**n**	**%**
**Gender**	Boys	475	80.1%
	Girls	118	19.9%
**Age**	4 years old	128	21.6%
	5 years old	114	19.2%
	6 years old	78	13.2%
	7 years old	58	9.8%
	8 years old	60	10.1%
	9 years old	53	8.9%
	10 years old	53	8.9%
	11 years old	49	8.3%
**Diagnoses**[a]	Autism spectrum disorder and no other disease	492	83%
	Food allergies	39	6.5%
	Food intolerance	35	5.9%
	Down's syndrome	9	1.5%
	Eating disorders[b]	1	0.1%
	Attention deficit hyperactivity Disorder	16	2.6%
	Others diagnoses[c]	30	5.0%
**Brazilian regions**	Midwest	149	25.1%
	Northeast	99	16.7%
	North	33	5.6%
	Southeast	222	37.4%
	South	90	15.2%
**Housing area**	Urban area	560	94.4%
	Rural area	33	5.6%
**People living in the same house**	2	53	8.9%
	3	209	35.2%
	4	208	35.1%
	5	84	14.2%
	6	26	4.4%
	7	7	1.2%
	8	5	0.8%
	10	1	0.2%

[a] Children may have one or more diagnoses. [b] Anorexia/bulimia/pediatric eating disorder. [c] Such as other syndromes, cerebral palsy, epilepsy, and others.
